# Antecedents of Elderly Home Residency in Cognitive Healthy Elders: A Qualitative Study

**DOI:** 10.5539/gjhs.v5n2p200

**Published:** 2012-01-21

**Authors:** Abdolhosein Emami Sigaroudi, Nahid Dehghan Nayeri, Hamid Peyrovi

**Affiliations:** 1 Tehran University of Medical Sciences (TUMS), School of Nursing and Midwifery, Tehran, Iran; 2 Social Determinants of Health (SDH) Research Center, School of Nursing and Midwifery, Guilan University of Medical Sciences (GUMS), Guilan, Iran; 3 Nursing and Midwifery Care Research center, School of Nursing and Midwifery, Tehran University of Medical Sciences, Tehran, Iran; 4 Center for Nursing Care Research, Department of Critical Care Nursing, School of Nursing and Midwifery, Tehran University of Medical Sciences, Tehran, Iran

**Keywords:** elderly home, residence, predictor, thematic analysis

## Abstract

Admission to elderly homes brings with itself fear of losing home and many other mental consequences to both the elder and family members. The aim of this study was to explore the experiences of elders about factors influencing living in the elderly home. Using a qualitative design, 14 elder people living in elderly homes were chosen through purposive sampling based on defined criteria. Semi-structured interviews were held with the participants and field noting was taken during the interviews. Collected data was analyzed using thematic analysis, which resulted in the development of three main themes: “basic Predictors”, “self-care crisis”, and “role crisis”, which were consisted of a couple of subthemes. We conclude that nurses to better care in community and elderly homes, need to know that living in elderly homes has universal predictors; Of course their weight and position are different on different contexts.

## 1. Introduction

Improvements in quality of life, healthcare services, and life expectancy over the world have increased the number of people reaching elderliness in many countries (ECLAC, 2012; Farage, 2012). Although developed countries have experienced it, demographic changes in other countries have given rise to more considerable increase in the population of elders since 2010 (Kim et al., 2009). The overall number of elderly people around the world is increasing. In Iran, the overall population growth is 1.29%, but the growth rate for elderly people has been reported to be more than 3 percent (Iran Statistic Centre, 2012).

Increase in the population of elders has created challenges in economic, social and health aspects in the 21^th^ century. Along with population ageing, more long-term care services are required to be provided to elders (Rechel, Doyle, Grundy, & McKee, 2009). Elderly people need health care services more than other age groups (Denton, 2010). While many families are unable to provide care to their elder family members at own homes, the need for providing care to elders in elderly homes has become inevitable (Salarvand & Abedi, 2008).

Similar to other countries, the request for admission of elderly people to elderly homes has a rising trend in Iran (AdibHajbaghery & Aghahoseini, 2007). In this respect, until September 2007, about 270 round-the-clock elderly homes affiliated to the Iranian Welfare Organization were providing care to about 12000 elders (State Welfare Organization of Iran, 2011).

Moving to elderly homes has been reported to be a bitter experience. It not only confronts the elderly people with a change in their initial living places, but also makes them to deal with social and support networks and daily life routines (Salarvand & Abedi, 2008).

Although some studies have described factors affecting putting elderly people in elderly homes (Gaugler, Duval, Anderson, & Kane, 2007; Luppa, Luck, Matschinger, Konig, & Riedel Heller, 2010), no qualitative study has been conducted to explore the experiences of elderly people on factors resulting in putting elderly people in elderly homes. Improving our knowledge on the factors from elderly people's perspectives is of great significance to facilitate their transition and adaptation to the new living condition.

## 2. Background on Elderly Homes in Iran

Elderly home in Iranian mind and culture has negative connotations. Most of the families believe escrowing elderly to elderly home is due to lack of sense and apathy. In other words, putting the elders in elderly home is a stigma. It is rooted in our culture and sense of belonging. For example, in the Koran, God says: “If one of them [parents] or both of them reached their old age while staying with you, do not utter a word of disrespect such as “Oh” nor annoy them, but address them in terms of honor and kindness and be humble out of compassion and pray” (Rejeh, HeraviKarimooi, & Vaismoradi, 2011). In most cases, Iranian families are on the dilemma that whether or not put elderly in elderly homes. Now, going to the elderly home is requirements of urban life and the plight of found; thus, 10 percent of elderly people are living in elderly homes (Ebtkar, 2011).

## 3. Aim

The aim of this study was to explore the experiences of elders about factors influencing living in the elderly home.

## 4. Methods

A qualitative design was used to collect and analyze data.

### 4.1 Setting and Participants

This study was conducted from April 2011 to April 2012 through interviewing with 14 elderly people (5 females and 9 males) who were living in elderly homes. For maximum variation in sampling, the participants were selected from both private and public elderly homes.

The elders were selected using purposive sampling with the following inclusion criteria:

1) Being healthy in both general and cognitive aspects based on the Mini Mental State Examination;
2) Aged above 60 years
3) Able to speak Persian fluently
4) Willing to express experiences on moving to the elderly home
5) Having at least 6 months experience of living in the elderly home

### 4.2 Data Collection

Semi-structured interviews were held with the participants and field noting was taken during the interviews. The main foci of the interviews were: Will you please tell me, why you are living in the elderly home? How did you come here? What were your problems for stay at home?

Probing questions were also asked to follow the participant's thoughts and bring elucidation to their responses.

The sampling process continued until data saturation was reached where no new data was available to add to the variation of themes and subthemes (Tuckett, 2004). After obtaining informant consent and coordinating, interviews performed in a quiet environment in one of the rooms that was allocated for this purpose.

According to the participant's physical health, the duration of each interview varied between 36 and 72 minutes on average. The interviews sessions were repeated with those elders who interrupted the interview sessions and liked to be interviewed in future sessions; Finally 17 Interview sessions were held.

### 4.3 Ethical Considerations

This study was approved by the ethical committee of affiliated to Tehran University of Medical Sciences. The objective and method of the study was explained to the participants. The participants were informed that participation in the study was voluntary and they could withdraw from the study any moment without any penalty. In addition, the participants were asked to sign the written consent form for participating in the study and recording their voices. Moreover, they were ensured that their information and audio files would be kept confidential.

### 4.4 Data Analysis

Thematic analysis was used to analyze data. Thematic analysis was a process in which the central themes of the phenomenon under study are explored and described. This process claimed to be as exploring the themes through the scrutinizing and revising the data (Rice & Ezzy, 2001; Braun & Clarke, 2006).

To analyze data, the coding process was conducted without following any predefined framework or convention. In this respect, the researcher applied an interpretive thematic analysis approach through a constant forward and backward movement in the dataset. The analysis process was initiated through concentration on conceptual patterns and interesting topics in the data concurrently with data collection. The interviews were transcribed verbatim and initial codes were generated. Data analysis was finished with developing themes and subthemes.

## 5. Trustworthiness

In this study, brief reports of the transcribed interviews with the initial codes were provided to the all participants as member checking to confirm that the researchers were presenting their real world. The research team independently coded and categorized the data and, compared their findings. In cases of disagreement, further discussion was held to reach consensus. For transferability, an audit trail of the data analysis process was provided.

## 6. Findings

The average age of the participants was 69.9 years (SD =7.9). Their residence duration in the elderly home was 19.4 months (Mean =19.4, SD = 7.8).

Three main themes emerged during data analysis: “basic Predictors”, “self-care crisis”, and “role crisis”, which were consisted of a couple of subthemes. The first theme consisted of four subthemes: “feeling of empty home”, “integrity disorder”, “social crisis creators”, and “economic pressure”. The second theme was consisted of “capability drop” and “rise of demands” as its subthemes. Finally, the third theme was consisted of the following subthemes: “change in the structural role” and “change in the functional role”.

### 6.1 Basic Predictors

One of the main themes that emerged from participant's experiences is the “basic Predictors” which includes the themes “feeling of empty home” “integrity disorder” “social crisis creators” “economic pressure” that each represent reason for reside in elderly home and participants experience at least one of them. For this and because other main themes are also affected by this theme, it is called “basic Predictors”.

#### 6.1.1 Feeling of Empty Home

One of the main Predictors of residence in the elderly home, as mentioned by the participants, was the feeling of loneliness at home. This subtheme had, in turn, the relevant subdivisions as the absence of companion due to divorce or death of the spouse, offspring's marriage, and leaving the home. In some cases, children had left the home due to working issues or marriage and the elder people had been left alone. A 64-years old man living in a semi-public elderly home talked about reasons for his residence as:

*“My wife died and I was left with three daughters and one son. My daughters got married and my son finished his military service and is at work. If my wife were alive, she would not let them bring me here. She madly loved me. Compassion is a real good thing; my daughter has her own problems and cannot take care of me. She has her own life and kids”*.

#### 6.1.2 Integrity Disorder

This subtheme was related to structural or functional defect, and disorder in physical and psychological aspects. Although some elderly people were used to living alone, they moved to the elderly home due to integrity disorder. Elderly dysfunction or impairment strongly forced him to reliance and stay in elderly home. They stated that they were ill and they suffered from structural or functional disorders in one of their body parts. A 74-years old lady living in semi-public elderly home stated:

*“My husband died and I remarried by the help of one of my relatives. I found that I could not do my own chores and take care of my husband's affairs. My husband's kids suggested us [my husband and I]to return and live with our own kids. It was not possible for my kids to take care of me and I was not even able to stand on my feet. I was not even able to do my daily livings activities. My husband's kids tricked me, brought me here, and left me alone”*.

An 80-years old lady living in a private elderly home also stated:

*“My husband died once my kids were too young. I used to live alone for 17 to 18 years. My only daughter lives in Sweden. I was disabled and due to stroke I was unable to do my own activities. One of my kids was martyred in the war. The other two boys of mine were too busy and could not take care of me”*.

About impaired social and mental health, a 62-years old lady living in a private elderly home mentioned:

*“I divorced and was alone and had neurological problems. My only son asked me to marry a man who was suffering from cancer. Actually, I did not know about that and he [man] died soon. My son was with his father in Tehran and I was alone. I did not have energy to do my activities, and take care of myself; Thus, I asked him to bring me here”*.

#### 6.1.3 Social Crisis Creators

Based on participant's experiences, addiction, divorce, and lack of support systems, the dependency of elders to elderly homes triggered. A 65-years old man living for two years in a public elderly home, declared:

*“Due to addiction, I lost my job and my wife and kids left me. All my friends are retired and are living in welfare. But I have not minimum social welfare and life is difficult for me. But this situation, asking me what happened to me, is like a complex to me”*.

Moreover, lack of support systems forced some elders to move to the elderly home. The previous participant continues his remarks as: “Outside of the elderly home I had no hope. I had no place to live. Someone suggested me to come to the elderly home. To do so I had nobody to help me. Thus, I wrote myself a letter to the courthouse and told them winter is coming and I have nobody and no place to live. They wrote me back and also sent a letter to welfare organization and I am here now.”

#### 6.1.4 Economic Pressure

The participants claimed that low income and increase in living expenditures led to their admission to the elderly home.

A 61-years old lady living in a semi-public elderly home, who had no definite income, mentioned:

“After my husband death, I used to work and take care of myself, but my physical condition got worse and my leg paralyzed due to stroke. If I could hire someone to do my job, I preferred to live in my home. My family could afford 1500 000 Rials but I still needed another 1500 000 Rials to hire someone to take care of me. I don’t have that money and I have to bear the situation.”

### 6.2 Self-Care Crisis

Those elderly people, who did not have a place to live, reluctantly chose to live in the elderly home. Self-care crisis was created when the elder's physical ability was deteriorated and their demands increased.

#### 6.2.1 Capability Drop

Some elderly people were living at their own houses even after the death of their spouses, and experiencing health problems. However, by gradual decrease in their physical abilities, they were no longer able to live by themselves. An 80-year old man who was brought to the elderly home by the police mentioned:

“I am 80 years old now and I have no power, no money and nothing else. If I would not be admitted here [the elderly home], I was certainly dead by now.”

#### 6.2.1 Demands Rise

The participants experienced the deterioration of their physical abilities. In such a situation, they were required to take care of themselves. As they were not able any more to do so, they preferred to be taken care in elderly homes.

An 87-year old male living in a private elderly home remarked:

“After my wife's death, I used to live alone until I got sick. I could not control my urination and had to wear diaper. I was not able to take care of myself. I had to do more jobs such as cooking and washing my clothes myself.”

### 6.3 Role Crisis

Following basic Predictors and self-care crises, the participant's role structure and role performance were affected. In this respect and from participant's statements, some of their structural roles such as paternal, maternal, and marital were eliminated. Moreover, they were not able to meet expectations and do their defined duties. These affected the function of the elders, so they were brought to the elderly home by the family, and police.

A 65-years old man who enjoyed reading books and living in a public elderly home was willing to marry again, if someone would accept his condition and afford his expenses. He mentioned:

“I was a bus driver. I missed my children in a car accident. In that accident my leg was injured and I was not able to drive anymore. Then my husband also died. Paying the rent of house was difficult for me. It was not possible for me to live in the society by abiding the social rules.”

## 7. Discussion

This study described the experiences of elderly people on factors influencing living in the elderly home. One of the emerging concepts in this study is “Feeling of empty home” that contains loss of family members, especially spouses and children. Many studies have found that having family members and receiving informal care lead to decrease of the elderly home entry (Miller, 2010; Nihtila & Martikainen, 2008; Charles & Sevak, 2005; Losasso & Johnson, 2002; Van Houtven & Norton, 2004; Waidmann & Thomas, 2003). Additionally, some of these studies found losing the spouse and child, and living alone provoked elderly people to move to the elderly home. Nihtila & Martikainen (2008) reported that elderly people who lost their spouses were more prone to be put in the elderly home. In another study conducted by Nihtila & Martikainen (2008), the absence of spouse or child was reported to be the predictor of living in the elderly home (Miller, 2010). Many of Iranian families believe remarriage is not good, especially in the elderly. However, in recent years this view has changed and remarriage, particularly for men, is better acceptable. But widow's remarriage is not so good. Anyway, remarriage's negative effects on adaptation and life arrangement are apparent.

An important issue is that, in addition to the fact that putting in elderly home is a stigma, health delivery system does not provide sufficient services to support elders in their own homes and communities. Thus families are facing with problems in elders care, including effect on the mind, body and Job. While in elderly homes (especially semi-public elderly homes) most of services are free, this can be because of difference in the elder's affairs organization responsible in elderly homes and community. Thus, although many studies have confirmed the content of this concept, in Iranian society with different family and health system structures, the effect of these issues is more and the loss of husband and children to be more problematic.

Additionally, it is believed that elderly people are seeking for a place in which they can experience more social contact with others (Salarvand & Abedi, 2008). Also, it is noted that the feeling of “empty house” experienced as either situational or developmental by the elderly people, led to moving elders to the elderly home. The marriage of elders is regarded as an unpleasant or even offensive phenomenon in Iranian society, which magnifies the importance of former problem even more. As a result, the elder has to deal with both death / divorce of his / her spouse and loneliness. Loneliness is experienced when significant social interactions are lost (Heravy, Rejeh, Foroughan, & Vaismoradi, 2012).

Another basic Predictor for living in the elderly home was the integrity disorder. Integrity refers to wholeness as a human being. It means that you have developed in to a person whose thoughts, words, and actions are congruent, and therefore do not conflict with each other (Wikipedia, 2012). Once a chronic illness or disability happens to elderly people, it is no longer possible for them to live by themselves (South Australia Dept of Health, 2009). Ageing may be accompanied with physical disabilities, being widow(er), and health deterioration, which can force the elderly people to change their life trend (Sarma & Simpson, 2007). Luppa, Luck, Matschinger, Konig, & Riedel Heller (2010) believe that physical illnesses and disabilities are among the Predictors of residency in the elderly home. The findings reported in the study of Hallis (2004) show that the feeling of decreased health status makes the elderly people prone to live in the elderly home.

Based on participant's experiences, divorce and addiction categorized as the creators of social crisis in this study can accelerate residency in the elderly home since they induce loneliness and social isolation. In a study conducted by Haghgu (2000), it was reported that divorce could result in loneliness in elderly people and it, by itself, is a factor affecting their transition to the elderly home. However, this factor has not been reported by any other similar study. In Iran, addiction involves stronger isolative consequences and addict people who are even able to live in the society are isolate merely by their addiction. Moreover, due to the high expenses of living in private elderly homes they have to move to semi-public elderly homes. It is also worth to mention that divorce is a destroying phenomenon in the Iranian society, and is considered as a big failure and stigma. In Iran, marriage is the best and only legitimate way to address some of the basic needs of human. Thus despite the difference in today's society, in the past generations (elders), divorce in cultural and ideological view is obscene and a big failure. So the experience of divorce on elderly in addition to the loss of power and increased dependence is more destructive. Thus post-divorce life is problematic.

Another factor detected through this study as a Predictor for the transition of elderly people in the elderly home was economic problems. Owing to several factors, elderly people have higher costs of health and living, which can threaten their independent life. Abedi and Salarvand (2008) mentioned the lack of financial resources for managing an independent life as the factor motivating elderly persons to live in elderly homes. In addition, in European countries, marital status and economic status of the family were among factors that affect on health condition and level of life satisfaction (Kalogirou & Murphy, 2006) and to be a resident of the elderly home (Salarvand & Abedi, 2008).

In the Iranian society, in the case of having sufficient financial resources, elderly people can live in their own houses and those with less integrity disorders but without economic support face serious self-care problems and need to live in the elderly home. This is related to loss of services for supporting elders in home or community, while those are defined in elderly homes.

Some participants in this study mentioned self-care crisis as a Predictor for their residence in the elderly home. They stated that diminishing their ability and rising demands led them to self-care crisis, which in turn forced them to live in the elderly home. Once mental and physical abilities of elderly people reached to the point that family or social supports cannot meet their needs, elders are forced to reside in elderly homes (Sarma & Simpson, 2007). In - Iranian healthcare system the role of community health nurse is not clearly defined, which makes elderly people dependence even more.

Based on findings of this study, role crisis was among factors contributing to elder's living in the elderly home through a change in their role's structures and functions. One of the main structural role changes during the elderly period occurs in matrimonial and parental roles, which causes a decrease in the individual control over his/her life. In general, elderly people are limited in their communication and their health status is degraded by ageing (Pasha, 2007). Bernston (2000) believes that elderly people are prone to lose their social roles such as parental, matrimonial, and occupational ones, which leads to their isolation. It is closely related to a change in the physiologic changes of individuals.

Furthermore, it has been theorized that a role change results in a lack of dominance feeling and depression symptoms (Krause, 1992). The lack of dominance feeling consists of five concepts including lack of power, isolation, frustration, losing the meaning, and anarchism (Van Willigen, 2000). Social roles help the individual to adjust with changes created in the life (Kritsotakis, Koutra, & Zografakis, 2012), and elderly people are less able to live in the society due to losing their former active roles. This phenomenon is of particular significance in Iran where in some cases the retirement age reaches the middle-age period. This leads to the above mentioned consequences such as a lack of power and isolation, which threatens elder's health.

## 8. Conclusion

In this study it was found that, certain problems induced elders to reside in elderly homes. Although antecedent factors are global, because of the people's belief and culture differences in societies, the effects (weight) of these factors may vary. However, the problems in many cases are inevitable, their effects can be modified and the age of onset can be delayed. Therefore, those involved in the elderly affairs, particularly community health nurses need to learn Predictors of residence in the elderly home and apply them in care planning and management of elderly in the community. Furthermore, the results of this study will help community health nurses and elderly home workers to understand the resident needs for care plan and try to resolve it.
